# Obesity Affects β2 Adrenergic Regulation of the Inflammatory Profile and Phenotype of Circulating Monocytes from Exercised Animals

**DOI:** 10.3390/nu11112630

**Published:** 2019-11-02

**Authors:** Isabel Gálvez, Leticia Martín-Cordero, María Dolores Hinchado, Alberto Álvarez-Barrientos, Eduardo Ortega

**Affiliations:** 1 Grupo de Investigación en Inmunofisiología, Departamento de Fisiología, Facultad de Ciencias, Universidad de Extremadura, 06071 Badajoz, Spain; igalvez@unex.es (I.G.); mhinsan@unex.es (M.D.H.); 2 Instituto Universitario de Investigación Biosanitaria de Extremadura (INUBE), 06071 Badajoz, Spain; leticiamartin@unex.es; 3 Grupo de Investigación en Inmunofisiología, Departamento de Enfermería, Centro Universitario de Plasencia, Universidad de Extremadura, 10600 Plasencia, Spain; 4 Servicio de Técnicas Aplicadas a la Biociencia (STAB), Universidad de Extremadura, 06071 Badajoz, Spain; alalvarez@unex.es

**Keywords:** β2 adrenergic receptors, terbutaline, obesity, exercise, monocytes, inflammation, cytokines, Ly6C, iNOS, arginase

## Abstract

Anomalous immune/inflammatory responses in obesity take place along with alterations in the neuroendocrine responses and dysregulation in the immune/stress feedback mechanisms. Exercise is a potential anti-inflammatory strategy in this context, but the influence of exercise on the β2 adrenergic regulation of the monocyte-mediated inflammatory response in obesity remains completely unknown. The first objective of this study was to analyze the effect of exercise on the inflammatory profile and phenotype of monocytes from obese and lean animals, and the second aim was to determine whether obesity could affect monocytes’ inflammatory response to β2 adrenergic activation in exercised animals. C57BL/6J mice were allocated to different lean or obese groups: sedentary, with acute exercise, or with regular exercise. The inflammatory profile and phenotype of their circulating monocytes were evaluated by flow cytometry in the presence or absence of the selective β2 adrenergic receptor agonist terbutaline. Exercise caused an anti-inflammatory effect in obese individuals and a pro-inflammatory effect in lean individuals. β2 adrenergic receptor stimulation exerted a global pro-inflammatory effect in monocytes from exercised obese animals and an anti-inflammatory effect in monocytes from exercised lean animals. Thus, β2 adrenergic regulation of inflammation in monocytes from exercised animals seems to depend on the inflammatory basal set-point.

## 1. Introduction

In recent decades, obesity has become a worldwide problem of epidemic proportions, and it is typically defined as an abnormal accumulation of body fat or excess body weight for a certain height. The major cause of obesity is a chronic positive energy balance, which takes place when energy intake is higher than energy expenditure. Thus, although it is a complex and multifactorial disease that results from a combination of genetic, epigenetic, and environmental factors, diet and physical exercise are generally the main factors involved in its etiology [1,2]. Obesity is strongly associated with increased mortality risk and the development of chronic comorbid conditions, especially a cluster of metabolic disorders known as metabolic syndrome (combination of obesity, hypertension, hyperglycemia, and dyslipidemia, with concomitant increased risk of cardiovascular events) [2,3]. It is also well accepted that obesity promotes chronic low-grade inflammation [4,5,6,7,8], a systemic condition characterized by increased systemic levels of some inflammatory cytokines (TNF-α, IL-1β, IL-6) among other inflammatory mediators such as C reactive protein (CRP) [9]. Obesity-induced low-grade inflammation exerts marked effects on metabolic pathways, playing a crucial role in the development of atherosclerosis and insulin resistance and all of the other characteristics associated with metabolic syndrome [9,10,11,12]. The trigger for this inflammation is uncertain, and the causal relationship between inflammation and these complications is not fully known [13]. Notably, local inflammation of adipose tissue contributes largely to systemic low-grade inflammation since adipocytes and resident macrophages are a key source of secreted pro-inflammatory factors into circulation [12,14].

Moreover, since obesity is also linked to altered hypothalamic–pituitary–adrenal axis (HPA) and sympathetic nervous system (SNS) function [15,16,17], anomalous immune/inflammatory responses in this condition take place along with alterations in neuroendocrine responses and dysregulation in immune/stress feedback mechanisms [17,18,19]. Adrenergic agonists such as catecholamines secreted by the SNS and the adrenal glands are involved in regulating both metabolism and most of the mechanisms of the immune response, including the innate response and the systemic and local release of inflammatory cytokines [20,21,22,23,24,25].

Different immune cell types, including monocytes, present β adrenergic receptors (ARs), and more specifically, β2 AR for catecholamines and other adrenergic agonists [26,27,28]. Many immune/inflammatory processes can be affected by adrenergic agonists via β AR-dependent mechanisms in immune cells [28,29,30], being able to induce both pro- and anti-inflammatory responses depending on several factors and conditions [23,31]. The effect of β2 AR activation on monocytes is usually anti-inflammatory and immunosuppressive, although under certain conditions they can result in pro-inflammatory effects [25,32]. Systemically, an excessive immune response or inflammatory state normally stimulates a negative feedback stress mechanism, which protects the organism from an “overshoot” of pro-inflammatory cytokines and other inflammatory mediators [21,33]. In obesity, monocytes are in a pro-inflammatory state, and β2 AR stimulation induces a shift towards an anti-inflammatory phenotype and activity profile, whereas it induces a shift towards a pro-inflammatory phenotype and activity profile in monocytes from lean individuals, thus being anti-inflammatory only in the case of an elevated inflammatory status [34]. Thus, high inflammatory basal set-points, like those found in chronic low-grade inflammatory conditions, can determine different effects of β2 AR stimulation on inflammatory responses.

Another factor that could influence the β2 adrenergic regulation of inflammatory processes in monocytes is exercise. It is currently accepted that the beneficial effects of regular exercise are exerted through its anti-inflammatory effects by increasing catecholamine levels (due to activation of the HPA axis and the SNS) and potentially decreasing the percentage of cells with an inflammatory profile [35]. However, although there has been some research regarding β adrenergic regulation in this context [36,37], it is still unclear exactly how exercise influences β2 AR-mediated inflammatory changes in immune cells, particularly monocytes. Bearing in mind that exercise itself can exert differential effects on the immune/inflammatory and stress responses in obese individuals and in lean ones [17,38], it is plausible to think that exercise could affect β2 adrenergic regulation of inflammation in obesity differently than in healthy individuals [25]. In fact, the influence of exercise (both acute and regular) on the β2 adrenergic regulation of the monocyte-mediated inflammatory response in obesity remains completely unknown.

Thus, the first objective of this study was to analyze whether the effect of exercise (both acute and regular exercise) on the inflammatory profile and phenotype of monocytes could be different in obese animals versus lean ones, taking into account their different basal inflammatory set-point. The second objective was to determine whether exercise could affect monocytes’ inflammatory response to β2 adrenergic activation in lean and obese animals, and thus if obesity could also affect monocytes’ inflammatory response to β2 adrenergic activation in exercised animals.

## 2. Materials and Methods

### 2.1. Animals and Experimental Design

C57BL/6J mice were housed and bred in the animal facilities of the University of Extremadura from stock originally obtained from Envigo (Huntingdon, UK). At eight weeks of age, 39 mice were randomly allocated (using a random number generator on a computer) to one of two diets until sacrifice 18 weeks later. To obtain an experimental model of obesity, one group (n = 19) (obese group) was placed on a high-fat diet (HFD) (260HF diet; SAFE, Augy, France) containing 36% fat (58.8% of the energy from fat). The other group (n = 20) constituted the healthy control group (lean group) and was placed on standard laboratory rodent chow (SD) (A04 diet; SAFE, Augy, France), containing 3.1% fat (8.4% of the energy from fat). After 10 weeks of the diet protocol, at 18 weeks of age, mice from both the lean and obese group were randomly allocated to either a sedentary group or an exercised group. The sedentary groups (lean sedentary group n = 14, obese sedentary group n = 13) did not perform any kind of exercise whereas the exercised groups (lean exercised group n = 6, obese exercised group n = 6) underwent a program of regular exercise for eight weeks, until the age of 26 weeks, when all mice were sacrificed and samples collected. Just before sacrifice, mice from both the lean and obese sedentary groups were randomly allocated to either continuing in the corresponding sedentary group (lean sedentary group n = 9, obese sedentary group n = 8) or an acute exercise group (acute exercise lean group n = 5, acute exercise obese group n = 5). Acute exercise groups performed an acute bout of exercise immediately before sacrifice.

In order to quantify individual food consumption, mice were housed individually in cages with free access to food and water throughout the study. Olfactory and visual contact between mice was possible all along the study to avoid potential harmful effects of isolation. The cages were kept in a temperature- and humidity-controlled room (temperature, 22 ± 1 °C; humidity, 60 ± 5%) and exposed to a 12 h light/12 h dark cycle. Body weight, nose-to-anus length, and food consumption measurements started the first week of the protocol (eight weeks of age) and continued weekly for the entire lifespan of each mouse. Food consumption was determined by weighing the total amount of food given at the start of each week and then subtracting by the amount of food remaining at the end of the week. At approximately 6.5 months of age, after 18 weeks of the diet protocol with the last eight weeks also including exercise protocol, blood samples were collected from anaesthetized animals. Blood collection occurred following a 12 h fast. All of the evaluated parameters were determined in each animal.

In the present work, firstly we evaluated the effect of regular exercise or of an acute bout of exercise on β2 AR expression and the inflammatory profile and phenotype of monocytes in obesity, as compared to the effect on lean animals. Secondly, we assessed, comparatively in both obese and lean animals, the influence of regular exercise or of an acute bout of exercise on the β2 adrenergic regulation of the inflammatory profile and phenotype of monocytes.

The study was approved by the Bioethics Committee for Animal Experimentation of the University of Extremadura (registry number 115/2015), in accordance with the ARRIVE guidelines and the National and European legislation for the protection of animals used for research.

**Figure 1:**
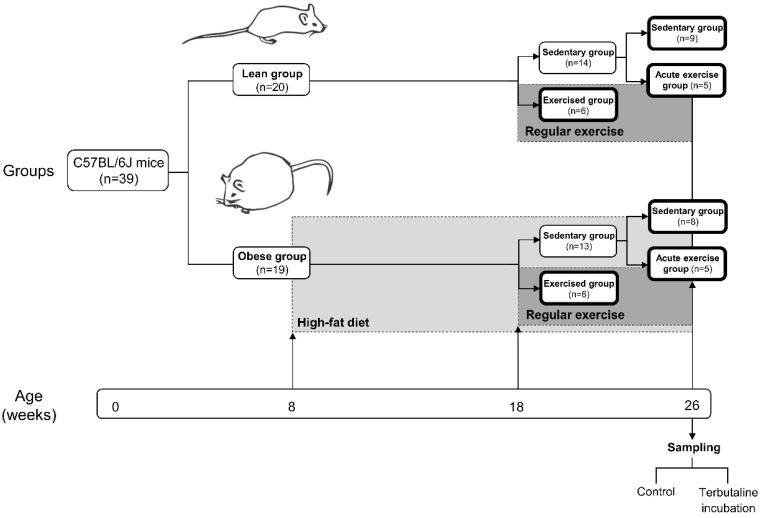
Schematic diagram of the experimental design of the study showing mice groups, dietary and exercise interventions, chronogram and sample treatment.

Figure 1 shows a schematic diagram of the experimental design of the study.

### 2.2. Exercise Protocol

The protocol of regular exercise began at approximately 18 weeks of age, after 10 weeks of the diet protocol. It was performed for 8 weeks, 3 days per week, in the active period of the animals (dark period, 11:00–23:00 h), starting always at 12:00 h. The regular exercise training consisted of running on a treadmill (model 800, IITC Life Science Inc., Los Angeles, CA, USA), with no slope, and with duration and intensity adaptation, progression, and maintenance phases. Exercise sessions progressed from 10 m/min for 10 min in the first week to 18 m/min for 45 min in the last week. This protocol of regular exercise is accepted to be able to induce physiological adaptations in mice [39,40]. Animals were sacrificed 72 h after the last training in order to avoid the evaluation of the acute effects of exercise.

The bout of acute exercise was also performed in the active period of animals (dark period, 11:00–23:00 h), starting at 12:00 h. It consisted of running on the treadmill for 5 min at 10 m/min followed by a progressive increase to 16 m/min for 35 min, with no slope. Animals were sacrificed and samples collected immediately after the session.

### 2.3. Anaesthesia and Whole Blood Collection

After a 12 h fast, mice were anaesthetized with an induction dose of 3%–5% isoflurane, and a maintenance dose of 1.5%–3% isoflurane. Then, whole blood was drawn from live, anaesthetized animals by cardiac puncture using heparinized syringes.

### 2.4. Fasting Glucose Levels and Lipid Profile Determination

A volume of 50 µL of whole blood was used for the determination of fasting glucose levels and lipid profile (total cholesterol [TC], high-density lipoprotein cholesterol [HDL-C], calculated low-density lipoprotein cholesterol [cLDL-C], and triglycerides [TG]) using the portable analytical device Lux (Biochemical Systems International Srl, Arezzo, Italy). Glucose levels were determined using reagent strips based on an electrochemical method (glucose oxidase method), while the results of the lipid profile were based on a reflectometry method.

### 2.5. Inflammatory Profile and Phenotype of Circulating Monocytes: Inflammatory Cytokines, Ly6C, Inducible Nitric Oxide Synthase, and Arginase-1 Expression Assays by Flow Cytometry

Blood was diluted in RPMI 1640 complete medium (L-glutamine and penicillin-streptomycin) (Thermo Fisher Scientific, Waltham, MA, USA) except FBS and distributed in 24 well plates. Cells were cultured with brefeldin A solution (1 μg/mL) (Thermo Fisher Scientific, Waltham, MA, USA), a protein transport inhibitor for the enhancement of the intracellular staining of cytokines, in the presence or absence of the selective β2 AR agonist terbutaline (1 μM) (Sigma-Aldrich, St. Louis, MO, USA). Plates were incubated at 37 °C in a 5% CO_2_ incubator for 5 h.

Then, samples were centrifuged at 300× *g* for 10 min. Supernatants were discarded, and pellets were resuspended in 600 µL of staining buffer, consisting of phosphate buffered saline (PBS) solution, 0.5% bovine serum albumin (BSA) (Thermo Fisher Scientific, Waltham, MA, USA), and 2 mM EDTA (Thermo Fisher Scientific, Waltham, MA, USA), plus 750 µL of Inside Fix reagent from Inside Stain Kit (Miltenyi Biotec, Bergisch Gladbach, Germany) for the fixation of cells for intracellular staining. Cells were incubated for 25 min at room temperature in darkness and agitation. After that, samples were centrifuged at 300× *g* for 5 min and pellets were resuspended in 300 µL of staining buffer; and kept at 4 °C overnight. Again, samples were centrifuged at 300× *g* for 5 min and then pellets were resuspended in 300 µL of Inside Perm reagent from Inside Stain Kit (Miltenyi Biotec, Bergisch Gladbach, Germany) for the permeabilization of cells for intracellular staining, and dispensed in a 96-well plate (50 µL per well).

Cells were incubated with the respective conjugated antibodies for the evaluation of the membrane expression of Ly6C (Anti-Ly-6C-PerCP-Vio700, Miltenyi Biotec, Bergisch Gladbach, Germany) and β2 AR (ADRB2 Polyclonal Antibody, Alexa Fluor 647 Conjugated, Bioss Antibodies, Woburn, MA, USA), as well as the intracellular expression of inducible nitric oxide synthase (iNOS) (iNOS antibody 4E5, Novus Biologicals, Centennial, CO, USA), arginase-1 (ARG-1) (ARG1 PE, Novus Biologicals, Centennial, CO, USA), monocyte chemoattractant protein-1 (MCP-1) (Anti-CCL2(MCP-1)-PE, Miltenyi Biotec, Bergisch Gladbach, Germany), TNF-α (Anti-TNF-α-FITC, Miltenyi Biotec, Bergisch Gladbach, Germany), IL-8 (CXCR1/IL-8 RA APC, Novus Biologicals, Centennial, CO, USA), IL-6 (Anti-IL-6-PE, Miltenyi Biotec, Bergisch Gladbach, Germany), IL-10 (Anti-IL-10-APC, Miltenyi Biotec, Bergisch Gladbach, Germany), and TGF-β (LAP PE-Cyanine7, Thermo Fisher Scientific, Waltham, MA, USA) in monocytes. First, iNOS antibody was incubated for 30 min at room temperature in darkness and agitation, and then cells were washed and incubated with Alexa Fluor 430 anti-mouse conjugated secondary antibody (Thermo Fisher Scientific, Waltham, MA, USA) for another 30 min. After another wash, the rest of antibodies were added and incubated for 20 min at room temperature, in the dark with shaking. Optimal concentrations of each antibody were established after titration. After the incubation and cellular fixation protocol, and subsequent cellular labelling with the conjugated antibodies of interest, plates were centrifuged, supernatants were removed, and 100 µL of Inside Perm reagent was added to each well. Finally, samples were analyzed by a flow cytometer (CytoFLEX S, Beckman Coulter Life Sciences, Indianapolis, IN, USA). A minimum of 5000 cells were acquired by well. Data were processed using the CytExpert software (Beckman Coulter Life Sciences, Indianapolis, IN, USA). Data were analyzed on monocyte population gated by FSC/SSC parameters.

### 2.6. Statistical Analysis

Values are expressed as the mean ± standard error of the mean (SEM). Results regarding β2 adrenergic stimulation with terbutaline are expressed and statistically analyzed in percentage change from baseline, giving “100” to the basal values (in the absence of β2 adrenergic stimulation). The normal distribution of the variables was checked using the Kolmogorov–Smirnov normality test, followed by Student’s *t* test for comparisons between two groups. The minimum significance level was set at *p* < 0.05. Statistical analyses were performed with GraphPad Prism 7.0 (GraphPad Software Inc., San Diego, CA, USA).

## 3. Results and Discussion

### 3.1. Weight Measurements, Dietary Intake, Fasting Blood Glucose, and Lipid Profile

Significant weight differences between the lean and obese groups began to be observed in the first weeks of the diet protocol, and these differences remained significant until the end of the intervention. As expected in our model of HFD-induced obesity, body weight at sacrifice was significantly higher in animals fed a HFD than in those fed a SD, in all groups: sedentary (*p* < 0.001), acute exercise (*p* < 0.01) and regular exercise (*p* < 0.01). After the exercise protocol, only the obese mice who performed regular exercise presented lower body weight than their corresponding sedentary group (*p* < 0.05).

In addition, fasting blood glucose levels as well as triglycerides, total cholesterol, HDL-C, and cLDL-C levels were significantly higher in obese sedentary mice than in lean sedentary mice. Both lean and obese mice performing regular exercise presented higher levels of HDL-C (*p* < 0.05) as well as lower cLDL-C (*p* < 0.05) and triglycerides (*p* < 0.05 and *p* < 0.001, respectively) levels than their corresponding sedentary group (Table 1). It is noteworthy that fasting blood glucose levels measured by our device and technique in blood obtained by cardiac puncture seem to be slightly higher than those usually reported in C57BL/6J mice (around 150 mg/dL in lean sedentary animals).

### 3.2. Influence of Obesity in the Effect of Exercise on the Inflammatory Profile and Phenotype of Circulating Monocytes

**Figure 2:**
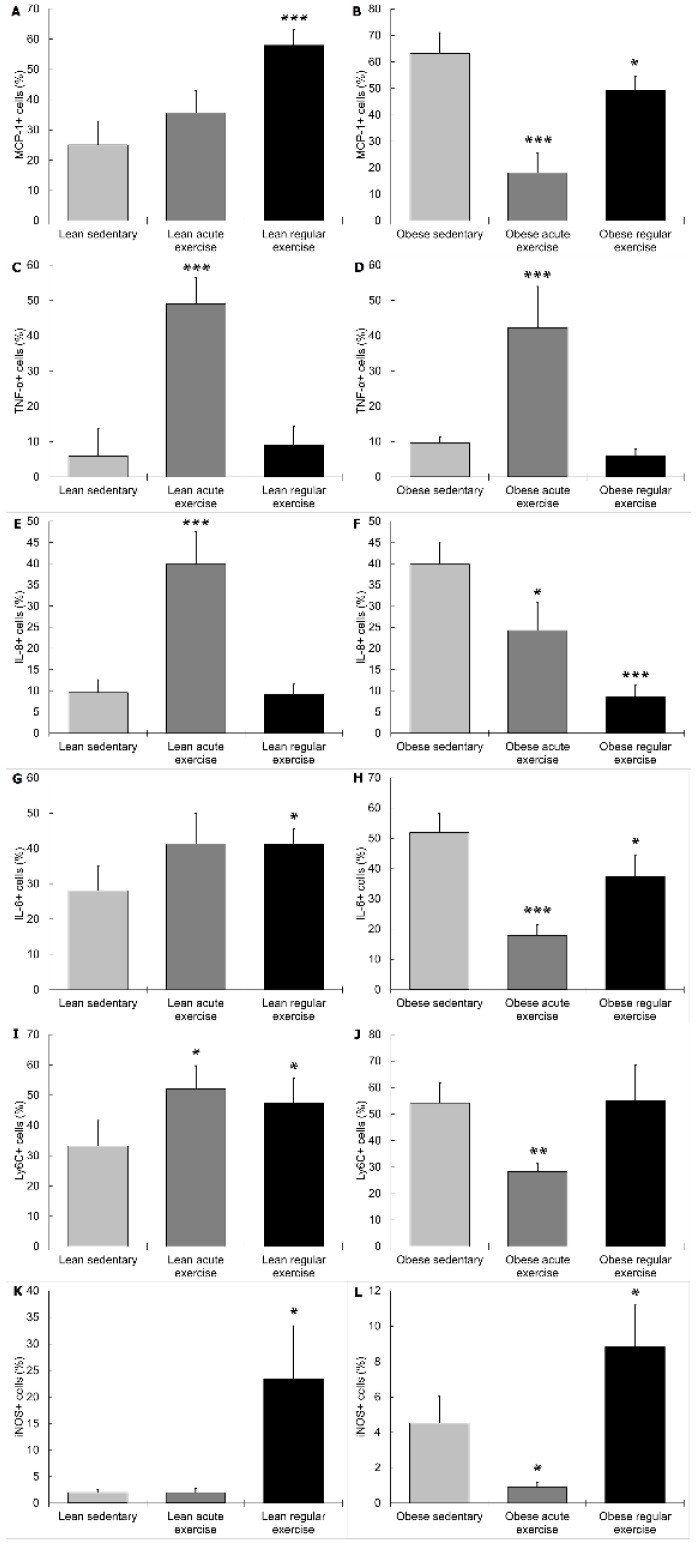
Effect of exercise on monocytes expressing pro-inflammatory cytokines (monocyte chemoattractant protein-1 (MCP-1), TNF-α, IL-8, IL-6) and pro-inflammatory phenotype (Ly6C, inducible nitric oxide synthase (iNOS)) in lean (sedentary n = 9; acute exercise n = 5; regular exercise n = 6) and obese mice (sedentary n = 8; acute exercise n = 5; regular exercise n = 6). (**A**,**B**): % MCP-1+ cells in lean and obese mice, respectively. (**C**,**D**): % TNF-α+ cells in lean and obese mice, respectively. (**E**,**E**): % IL-8+ cells in lean and obese mice, respectively. (**G**,**H**): % IL-6+ cells in lean and obese mice, respectively. (**I**,**J**): % Ly6C+ cells in lean and obese mice, respectively. (**K**,**L**): % iNOS+ cells in lean and obese mice, respectively. Columns show the mean ± SEM of independent assays performed in duplicate in each animal. * *p* < 0.05, ** *p* < 0.01, *** *p* < 0.001 vs. the corresponding sedentary mice group.

**Figure 3:**
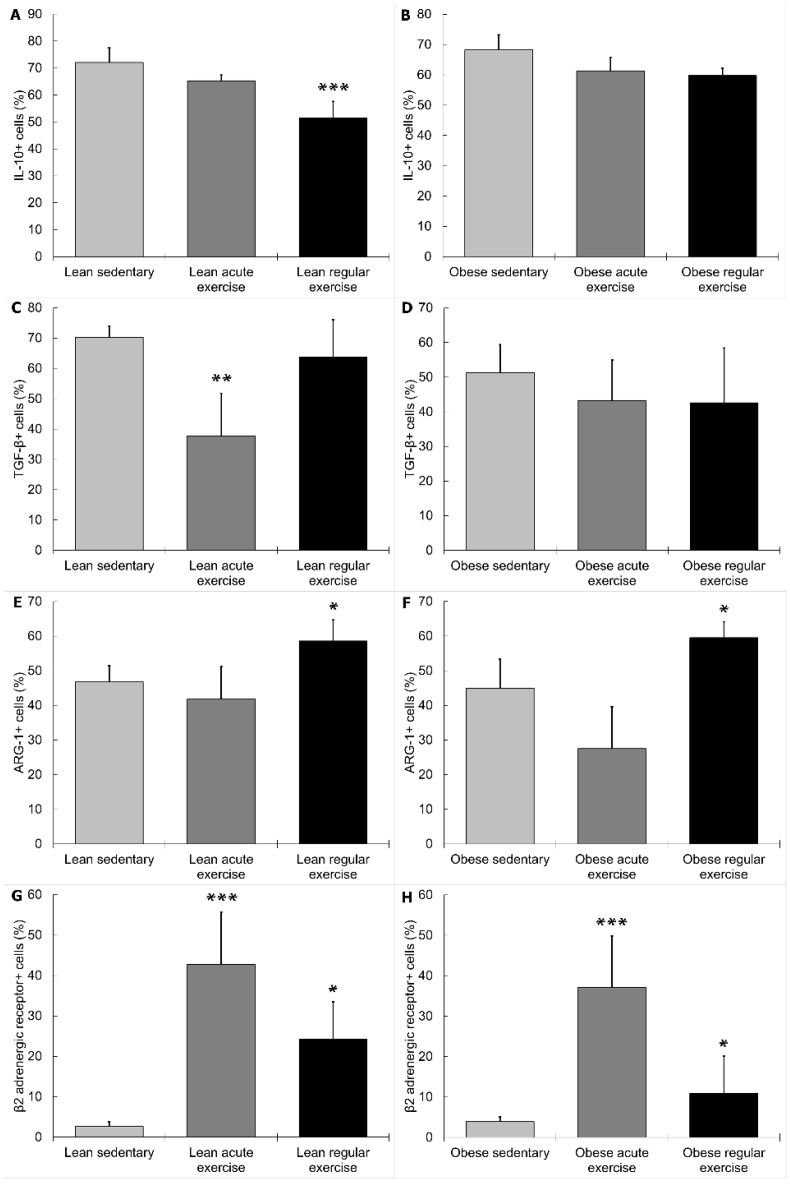
Effect of exercise on monocytes expressing anti-inflammatory cytokines (IL-10 and TGF-β), anti-inflammatory phenotype (arginase-1 (ARG-1)), and β2 adrenergic receptors in lean (sedentary n = 9; acute exercise n = 5; regular exercise n = 6) and obese mice (sedentary n = 8; acute exercise n = 5; regular exercise n = 6). (**A**,**B**): % IL-10+ cells in lean and obese mice, respectively. (**C**,**D**): % TGF-β+ cells in lean and obese mice, respectively. (**E**,**F**): % ARG-1+ cells in lean and obese mice, respectively. (**G**,**H**): % β2 adrenergic receptor+ cells in lean and obese mice, respectively. Columns show the mean ± SEM of independent assays performed in duplicate in each animal. * *p* < 0.05, ** *p* < 0.01, *** *p* < 0.001 vs. the corresponding sedentary mice group.

Circulating monocytes from lean sedentary animals performing the acute bout of exercise showed higher expression of TNF-α (*p* < 0.001, Figure 2C), IL-8 (*p* < 0.001, Figure 2E), and Ly6C (*p* < 0.05, Figure 2I), and lower expression of TGF-β (*p* < 0.01, Figure 3C) than those from lean sedentary animals without exercise. Monocytes from obese sedentary animals after the acute bout of exercise presented higher TNF-α expression (*p* < 0.001, Figure 2D); while MCP-1 (*p* < 0.001, Figure 2B), IL-8 (*p* < 0.05, Figure 2F), IL-6 (*p* < 0.001, Figure 2H), Ly6C (*p* < 0.01, Figure 2J) and iNOS (*p* < 0.05, Figure 2L) expression was significantly lower compared to obese sedentary animals without exercise.

Regular exercise stimulated MCP-1 (*p* < 0.001, Figure 2A), IL-6 (*p* < 0.05, Figure 2G), Ly6C (*p* < 0.05, Figure 2I), iNOS (*p* < 0.05, Figure 2K), and ARG-1 (*p* < 0.05, Figure 3E) expression, and reduced IL-10 (*p* < 0.001, Figure 3A) expression in lean mice compared to sedentary lean mice. In obese mice performing regular exercise, the percentage of monocytes expressing iNOS (*p* < 0.05, Figure 2L) and ARG-1 (*p* < 0.05, Figure 3F) was higher, while the percentage of monocytes expressing MCP-1 (*p* < 0.05, Figure 2B), IL-8 (*p* < 0.001, Figure 2F), and IL-6 (*p* < 0.05, Figure 2H) was lower than in obese sedentary animals.

It is well known that obesity is a low-grade inflammatory condition [4,5] and, moreover, our group has recently found that monocytes from obese mice present a pro-inflammatory profile and phenotype compared to those from lean mice [34], thus confirming the elevated inflammatory basal set-point of monocytes from obese individuals. As shown in results above, the effect of exercise on the monocyte-mediated inflammatory response was a global anti-inflammatory effect in obese animals, and pro-inflammatory or without changes in lean animals. This effect was especially notable for the cytokines MCP-1, IL-8, IL-6 and IL-10, and the marker Ly6C. These results are in agreement with the “bioregulatory effect of exercise” [38], which proposes that physiological responses to exercise can differ according to the inflammatory basal set-point. Thus, exercise can stimulate the inflammatory responses in healthy people, constituting an “alert state”, a positive immunophysiological adaptation against pathogenic attacks in a situation of vulnerability for the organism [41], whereas exercise can exert anti-inflammatory effects in individuals with an unhealthy inflammatory status [38]. This anti-inflammatory effect can contribute to the protective effect of exercise against chronic inflammation-associated diseases. Therefore, it can be proposed that exercise exerts differential effects on the inflammatory profile and phenotype of monocytes depending on their basal set-point. In line with this, previous studies from our laboratory showed that the regulation by exercise of the altered inflammatory status in genetically obese rats depends on each individual’s basal set-point, with anti-inflammatory effects mainly (or only) in those animals with an elevated inflammatory status [18].

Surprisingly, there were no significant changes in TNF-α, a very important cytokine in obesity, in lean nor obese animals performing regular exercise. After acute exercise, the percentage of monocytes expressing TNF-α was higher in both obese and lean mice compared to sedentary mice, a pro-inflammatory effect that might be physiologically explained in the context of innate immune response stimulation for the prevention of potential pathogen challenge during exercise-induced stress situations [38,41,42]. This phenomenon has also been observed with other pro-inflammatory cytokines in obesity [43]. Nevertheless, with regular exercise, an adaptation to exercise seems to occur, and TNF-α expression reaches similar values to those of sedentary animals. In addition, some differential anti-inflammatory effects of exercise (obese versus lean animals) seem to be more evident after an acute bout of exercise, as it can be observed in iNOS expression. The surprisingly increased iNOS+ monocytes after regular exercise in the obese animals, not following the global anti-inflammatory behavior, could also be a partial limitation to our interpretation of the global inflammatory effects.

The percentage of monocytes expressing β2 AR increased significantly in all exercise groups (acute exercise and regular exercise, both lean (*p* < 0.001 and *p* < 0.05 respectively, Figure 3G) and obese mice (*p* < 0.001 and *p* < 0.05, respectively, Figure 3H)) with respect to the corresponding sedentary groups. There are inconsistent results regarding the effect of exercise on β2 AR expression on immune cells, and monocytes in particular [44,45,46,47]. Most of them show an upregulation of the expression of this receptor in mononuclear cells [45,46] and monocytes [47] from healthy individuals after an acute bout of exercise. However, it was not clear if this response would be the same in obesity, most importantly in obese animals performing regular exercise. Our results showed that exercise modulates the expression of β2 AR in monocytes from lean and obese mice by significantly upregulating its expression, especially after acute exercise. However, the differential inflammatory responses to exercise in lean and obese mice do not seem to depend only on this modulation (since it is the same in lean and obese mice), but also in the different inflammatory basal set-point [34]. Moreover, catecholamine-mediated inflammatory effects of exercise are mediated not only by β2 AR but also by the other adrenoreceptors [48].

### 3.3. Influence of Obesity on the β2 Adrenergic Regulation of the Inflammatory Profile and Phenotype of Circulating Monocytes from Exercised Animals

The influence of obesity on the β2 AR-mediated modulation of inflammation in exercised individuals is still completely unknown and, to the best of our knowledge, the present study is the first one to perform a comprehensive assessment in monocytes in this context. In this study, we used terbutaline, a β adrenergic agonist that selectively stimulates β2 AR [49] and is commonly used in inflammation-related research [34,50].

**Figure 4:**
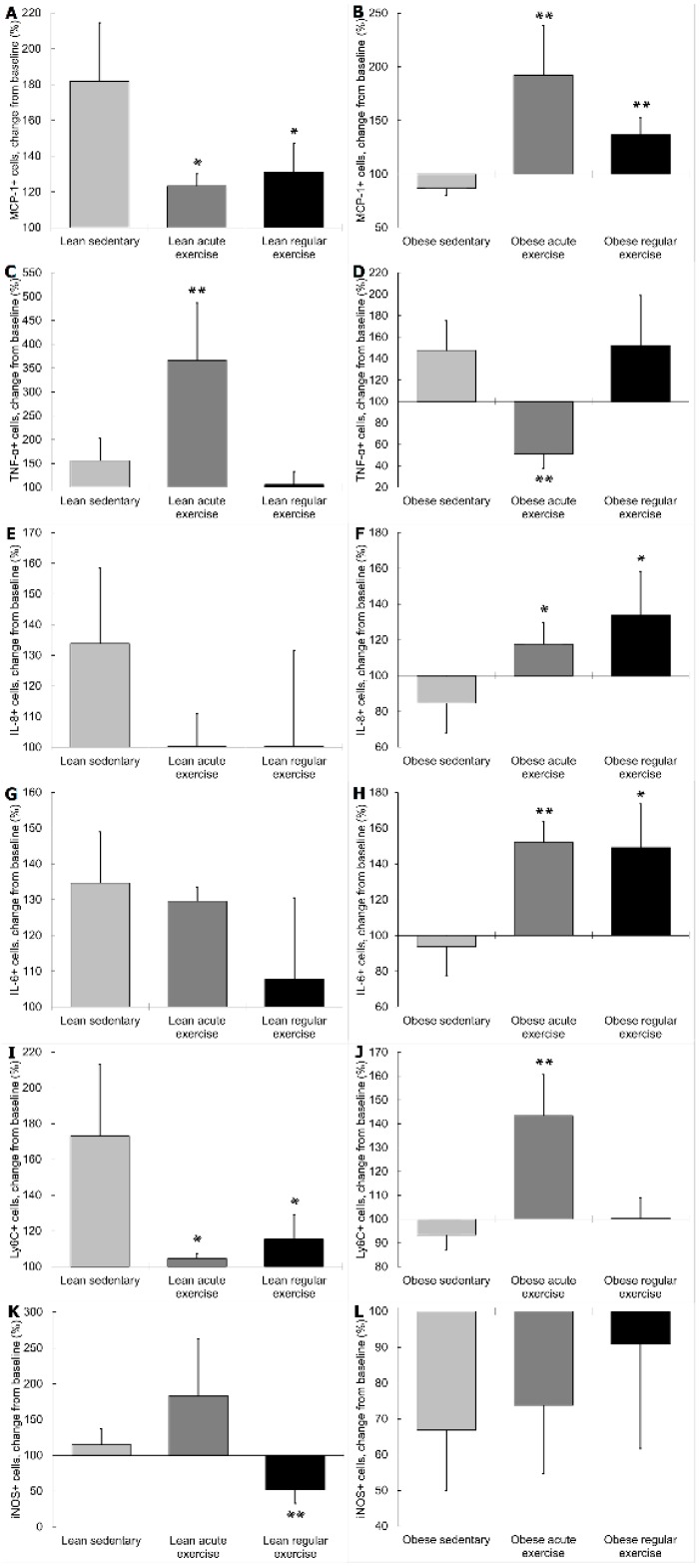
Effect of exercise on β2 adrenergic regulation of monocytes expressing pro-inflammatory cytokines (MCP-1, TNF-α, IL-8, IL-6) and pro-inflammatory phenotype (Ly6C, iNOS) in lean (sedentary n = 9; acute exercise n = 5; regular exercise n = 6) and obese mice (sedentary n = 8; acute exercise n = 5; regular exercise n = 6). Data are expressed as percentage change from baseline after β2 adrenergic stimulation with terbutaline (giving “100” to the basal values in the absence of terbutaline): (**A**,**B**): MCP-1+ cells in lean and obese mice, respectively. (**C**,**D**): TNF-α+ cells in lean and obese mice, respectively. (**E**,**F**): IL-8+ cells in lean and obese mice, respectively. (**G**,**H**): IL-6+ cells in lean and obese mice, respectively. (**I**,**J**): Ly6C+ cells in lean and obese mice, respectively. (**K**,**L**): iNOS+ cells in lean and obese mice, respectively. Columns show the mean ± SEM of independent assays performed in duplicate in each animal. * *p* < 0.05, ** *p* < 0.01 vs. the corresponding sedentary mice group.

β2 adrenergic activation increased monocytes expressing TNF-α in lean mice performing an acute bout of exercise significantly more than in monocytes from lean sedentary animals (*p* < 0.01, Figure 4C). Conversely, monocytes expressing TNF-α decreased in obese animals with acute exercise, a contrary effect to that found in obese sedentary mice (*p* < 0.01, Figure 4D). Regular exercise did not affect TNF-α expression of monocytes in response to terbutaline, neither in lean nor obese mice.

β2 adrenergic activation increased monocytes expressing MCP-1 and Ly6C in lean mice performing acute exercise significantly less (*p* < 0.05, Figure 4A,I) than in lean sedentary animals, whereas MCP-1 and Ly6C expression also increased in obese mice with acute exercise, but significantly opposed to the inhibition occurring in obese sedentary mice (*p* < 0.01, Figure 4B,J). With the performance of regular exercise, β2 adrenergic activation in monocytes from lean mice caused an increase in monocytes expressing MCP-1 and Ly6C significantly less (*p* < 0.05, Figure 4A,I) than in lean sedentary animals, whereas monocytes expressing MCP-1 increased in obese mice, contrary to the inhibition occurring in obese sedentary mice (*p* < 0.01, Figure 4B). β2 adrenergic activation in monocytes from lean mice performing regular exercise decreased monocytes expressing iNOS, contrary (*p* < 0.01, Figure 4K) to the increase occurring in lean sedentary animals.

In obese mice performing an acute bout of exercise or regular exercise, β2 adrenergic activation increased monocytes expressing IL-8 and IL-6, a significantly opposite effect (IL-8 *p* < 0.05, Figure 4F; IL-6 *p* < 0.01 acute exercise, and 0.05 regular exercise, Figure 4H) to that found in obese sedentary mice. No significant changes in these cytokines in response to β2 AR stimulation were found in exercised lean animals compared to sedentary ones.

**Figure 5:**
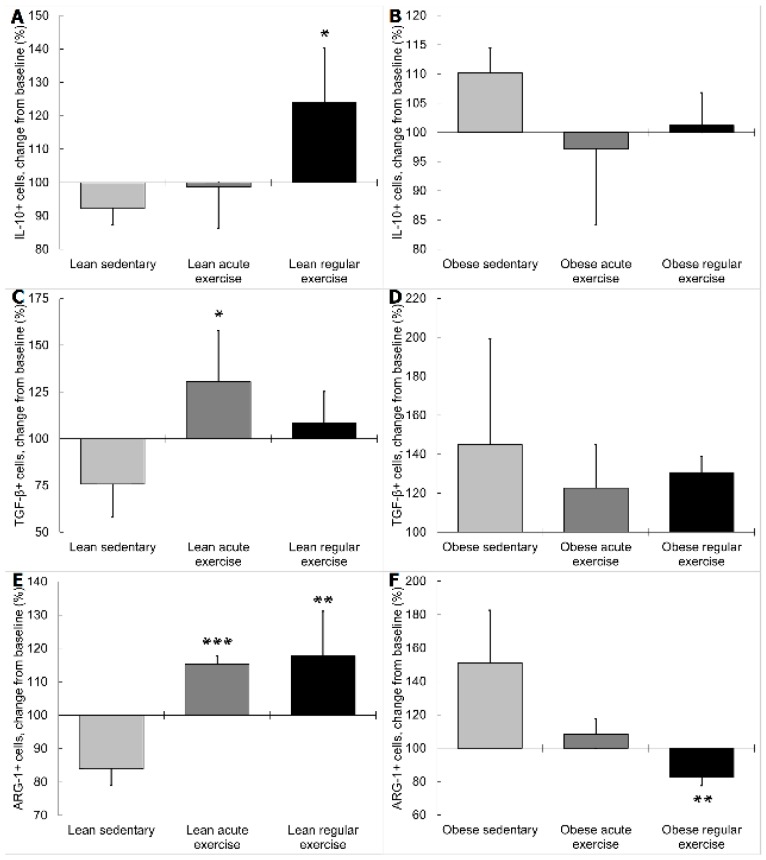
Effect of exercise on β2 adrenergic regulation of monocytes expressing anti-inflammatory cytokines (IL-10, TGF-β) and anti-inflammatory phenotype (ARG-1) in lean (sedentary n = 9; acute exercise n = 5; regular exercise n = 6) and obese mice (sedentary n = 8; acute exercise n = 5; regular exercise n = 6). Data are expressed as percentage change from baseline after β2 adrenergic stimulation with terbutaline (giving “100” to the basal values in the absence of terbutaline): (**A**,**B**): IL-10+ cells in lean and obese mice, respectively. (**C**,**D**): TGF-β+ cells in lean and obese mice, respectively. (**E**,**F**): ARG-1+ cells in lean and obese mice, respectively. Columns show the mean ± SEM of independent assays performed in duplicate in each animal. * *p* < 0.05, ** *p* < 0.01, *** *p* < 0.001 vs. the corresponding sedentary mice group.

In lean animals performing acute exercise, monocytes expressing TGF-β and ARG-1 increased in response to terbutaline, as significantly opposed (*p* < 0.05, Figure 5C; and *p* < 0.001, Figure 5E, respectively) to the inhibition observed in lean sedentary mice. In monocytes from lean animals with regular exercise, IL-10 and ARG-1 expression also increased with β2 adrenergic activation, opposite (*p* < 0.05, Figure 5A; and *p* < 0.01, Figure 5E, respectively) to the decrease observed in lean sedentary mice. However, in obese animals performing regular exercise, β2 adrenergic activation caused monocytes expressing ARG-1 to decrease, contrary (*p* < 0.01, Figure 5F) to the stimulation occurring in obese sedentary animals. Exercise did not affect iNOS, TGF-β, or IL-10 expression of monocytes from obese mice in response to terbutaline.

From these results, it is proposed that, globally, β2 AR stimulation has pro-inflammatory effects in monocytes from exercised obese animals and anti-inflammatory effects in exercised lean animals. These inflammatory effects were evident both in cytokine and in phenotype markers expression. Thus, β2 AR stimulation in both lean and obese exercised animals normally exerts contrary effects to those in sedentary animals [34], since exercise can switch the basal inflammatory status, especially in obese animals. This way, since exercise induces a shift towards an anti-inflammatory phenotype and activity profile in monocytes from obese individuals; whereas it induces a shift towards a pro-inflammatory phenotype and activity profile in monocytes from lean individuals (as shown and discussed in Section 3.2), β2 AR stimulation tends to revert these exercise-induced changes, with pro-inflammatory effects in monocytes from exercised obese animals and anti-inflammatory effects in monocytes from exercised lean animals. Thus, β2 adrenergic regulation of inflammation in monocytes from exercised animals seems to depend on the inflammatory basal set-point, only being anti-inflammatory when there is a high inflammatory status and pro-inflammatory or without changes when there is a healthy basal set-point, in the same way as in sedentary animals [34]. This could have important pathophysiological consequences when combining different potential anti-inflammatory strategies (both non-pharmacological and pharmacological) in individuals with inflammatory diseases that present a dysregulated basal inflammatory status.

To the best of our knowledge, this novel study is the first to evaluate β2 adrenergic regulation of the inflammatory profile and phenotype of monocytes in exercised obese individuals, and so it is not possible to discuss these results in relation to other similar investigations. In any case, these results are in agreement with the general concept of β2 adrenergic stimulation being anti-inflammatory in activated monocytes (with antigenic stimulation, for example with LPS) and pro-inflammatory in non-activated monocytes (resting conditions, in the absence of any stimulation) [25,32], which is particularly important in low-grade inflammatory diseases in which monocytes are in an activated state, such as obesity [34]. The immunophysiological relevance of these results, together with recent previous results from our group [34], is that β2 adrenergic activation of immune cells does not necessarily lead only to anti-inflammatory effects but exerts regulatory effects in order to restore the inflammatory homeostasis.

Surprisingly, only TNF-α responds to β2 AR stimulation in an opposite way to this general behavior both in lean and obese animals performing an acute bout of exercise. The percentage of monocytes expressing TNF-α increases significantly in lean and obese mice after acute exercise (Figure 2C,D). In response to β2 AR stimulation, lean animals performing acute exercise seemingly present a pro-inflammatory response (increase in TNF-α), whereas obese animals with acute exercise show an anti-inflammatory response (decrease in TNF-α) (Figure 4C,D). A possible explanation for this discrepant behavior could be a potential interaction with IL-6. β2 AR activation stimulates IL-6 expression in monocytes from obese animals performing acute exercise, and this could facilitate an inhibition of TNF-α expression, since IL-6 is a cytokine with inhibitory effects on TNF-α release by monocytes; which is the reason for attributing an anti-inflammatory effect to this cytokine in the context of exercise [9]. There is only one previous similar study in this context, which evaluated the effect of acute exercise on monocyte cytokine production in healthy volunteers. This study showed that TNF production by monocytes was downregulated during acute exercise, and that this effect was mediated by epinephrine through β2 AR stimulation [37], whereas our results show that healthy individuals present a higher percentage of monocytes expressing TNF-α in response to β2 AR stimulation after acute exercise. Therefore, further research on this topic seems to be necessary, including both the determination of synthesis and release of the inflammatory markers.

All of the results herein presented corroborate that it is not convenient to draw conclusions based solely on the results of one or two cytokines; on the contrary, it is important to take into account several cytokines and other inflammatory markers in order to properly interpret the global inflammatory response [51,52]. Even assuming the potential inaccuracies underlying any generalization, our conclusions are based on the global inflammatory behavior (which, in our opinion, is very important from an immunophysiological point of view); taking into account all the analyzed pro-/anti-inflammatory cytokines and markers, and the two modalities of exercise. In the light of these results, it is also crucial to always take into consideration potential adverse effects of inadequate physical exercise modalities in obesity, particularly if they are combined with anti-inflammatory pharmacological treatments via stimulation of β2 AR. This reinforces the idea that it is vital to individualize physical exercise prescriptions, depending on each individual’s physiological state and concomitant pharmacological therapies [25,38]. We believe the present study will contribute to the understanding of the influence of exercise in low-grade inflammation-related conditions on the neuroendocrine-immune responses, improving our knowledge of the adrenergic regulation of inflammation in different physiological and pathophysiological situations, and its potential therapeutic applications in this context. These novel results have neuro-immunophysiological relevance in the use of anti-inflammatory non-pharmacological (exercise) and pharmacological (β2 adrenergic agonists) strategies in obesity, and interactions or adverse effects in their potential combined use, given that β2 adrenergic agonists have been recently proposed as a potential pharmacological anti-inflammatory therapy in obesity and its complications [53]. Further studies should be focused on the intracellular mechanisms involved in this inflammatory response. It could be speculated that a different behavior in the changes of intracellular levels of cAMP must also be involved in the different responses to terbutaline according to the different inflammatory set-point in monocytes. Thus, it could be plausible to think that terbutaline-induced changes in monocyte intracellular levels of cAMP can also depend on the basal set-point of cAMP in this cell; and the increase or decrease in the intracellular levels of cAMP could be ultimately mediating these differential inflammatory responses to β2 adrenergic agonists.

## 4. Conclusions

Exercise induces a shift towards an anti-inflammatory phenotype and activity profile in monocytes from obese individuals, whereas it induces a shift towards a pro-inflammatory phenotype and activity profile in monocytes from lean individuals. β2 AR stimulation exerts a global pro-inflammatory effect in monocytes from exercised obese animals and anti-inflammatory effects in monocytes from exercised lean animals.

β2 AR stimulation tends to revert exercise-induced inflammatory changes in monocytes, with pro-inflammatory effects in exercised obese animals and anti-inflammatory effects in exercised lean animals. Thus, β2 adrenergic regulation of inflammation in monocytes from exercised animals seems to depend on the inflammatory basal set-point, only being anti-inflammatory when there is a high inflammatory status and pro-inflammatory or without changes when there is a healthy basal set-point.

## Figures and Tables

**Table 1:** Body weight, dietary and energy intake, and metabolic parameters in lean and obese mice, with or without acute or regular exercise.

	Lean			Obese		
	Sedentary	Acute Exercise	Regular Exercise	Sedentary	Acute Exercise	Regular Exercise
Body weight (g)	29.28 ± 1.17	30.15 ± 2.49	25.7 ± 1.27	42.28 ± 1.15 ***	40.37 ± 2.93	36.14 ± 2.98 ●
Dietary intake (g/day)	3.96 ± 0.22	4.16 ± 0.09	4.05 ± 0.06	2.68 ± 0.11 ***	2.62 ± 0.08	2.49 ± 0.03 ●●
Energy intake (kJ/day)	55.32 ± 3.15	58.16 ± 1.38	56.63 ± 0.86	62.01 ± 2.67 ***	60.50 ± 1.90	57.63 ± 0.71 ●●
Glucose (mg/dL)	218.90 ± 13.26	174.45 ± 32.06 ●	196.37 ± 25.53	311.50 ± 30.93 **	222.75 ± 23.12 ●	282.5 ± 27.85
Cholesterol (mg/dL)						
Total cholesterol HDL-C cLDL-C	103.69 ± 2.22 42.15 ± 2.93 50.75 ± 3.49	<99 ^†^	106.75 ± 2.90 51.75 ± 3.91 ● 39.4 ± 1.74 ●	172.70 ± 19.28 *** 59.70 ± 5.69 ** 88.83 ± 16.05 *	175 ± 41.82 55.5 ± 2.72 78 ± 12	178.12 ± 24.68 75.25 ± 4.39 ● 38.5 ± 1.5 ●
Triglycerides (mg/dL)	86.80 ± 1.86	88 ± 0.01	76.62 ± 0.73 ●	91.55 ± 1.99 *	98.75 ± 7	80 ± 1.43 ●●●

Data show the mean ± SEM of nine (lean sedentary group), eight (obese sedentary group), five (lean acute exercise group), five (obese acute exercise group), six (lean regular exercise group) and six (obese regular exercise group) independent experiments per animal. * *p* < 0.05, ** *p* < 0.01, *** *p* < 0.001 vs. lean sedentary mice group values; ● *p* < 0.05, ●● *p* < 0.01, ●●● *p* < 0.001 vs. the corresponding sedentary mice group values. HDL-C high-density lipoprotein cholesterol; cLDL-C calculated low-density lipoprotein cholesterol; ^†^ below limit of detection.
